# Thermal analysis, X-ray powder diffraction and electron microscopy data related with the production of 1:1 Caffeine:Glutaric Acid cocrystals

**DOI:** 10.1016/j.dib.2016.04.074

**Published:** 2016-05-12

**Authors:** Íris Duarte, Rita Andrade, João F. Pinto, Márcio Temtem

**Affiliations:** a Research Institute for Medicines (iMed.ULisboa), Faculty of Pharmacy, Universidade de Lisboa, Lisbon, Portugal; b R&D Drug Product Development, Hovione Farmaciência SA, Loures, Portugal

## Abstract

The data presented in this article are related to the production of 1:1 Caffeine:Glutaric Acid cocrystals as part of the research article entitled “Green production of cocrystals using a new solvent-free approach by spray congealing” (Duarte et al., 2016) [1]. More specifically, here we present the thermal analysis and the X-ray powder diffraction data for pure Glutaric Acid, used as a raw material in [1]. We also include the X-ray powder diffraction and electron microscopy data obtained for the 1:1 Caffeine:Glutaric Acid cocrystal (form II) produced using the cooling crystallization method reported in “Operating Regions in Cooling Cocrystallization of Caffeine and Glutaric Acid in Acetonitrile” (Yu et al., 2010) [2]. Lastly, we show the X-ray powder diffraction data obtained for assessing the purity of the 1:1 Caffeine:Glutaric cocrystals produced in [1].

**Specifications Table**

**Table:** 

Subject area	Pharmaceutical Technology
More specific subject area	Pharmaceutical cocrystals
Type of data	**Figures**
How data was acquired	**DSC using a TA Instruments Q1000 equipped with a RCS unit. XRPD using a D8 Advance Bruker AXS diffractometer with a Cu radiation source at 40 kV and 35 mA. SEM using a JEOL JSM- 7001F/Oxford INCA Energy 250/HKL operated in high vacuum and 15 kV.**
Data format	Analyzed
Experimental factors	**For the DSC, the sample weighted between 5–10 mg and was analyzed in a pinholed aluminum pan. The heating ramp was from 25 °C to 250 °C at 10 °C/min. For the XRPD total scans, the samples were measured over a 2θ interval from 3° to 70° with a step size of 0.017° and step time of 50 s. For the XRPD slow scans, the 2θ interval was from 10° to 14° with a step size of 0.017° and step time of 1500 s. For the SEM analysis, the sample was attached to adhesive carbon tape, previously fixed to an aluminum stub. The sample were left under vacuum for 2 h and then coated with gold/palladium.**
Experimental features	**The DSC shown corresponds to the total heat flow. The diffractogram of the 1:1 Caffeine:Glutaric Acid cocrystal obtained from the Cambridge Software Database (form II – EXUQUJ) is also presented. SEM images correspond to 50× and 200× magnifications.**
Data source location	Lisbon, Portugal
Data accessibility	Data is within this article

**Value of the data**

• The pure Glutaric Acid data can be used as a benchmark for other studies.
• The production of the 1:1 Caffeine:Glutaric Acid cocrystal (form II) by cooling crystallization validates the existing method [2], and can serve as a reference for other studies.
• The X-ray powder diffraction limit test is an alternative and simple method to estimate cocrystal purity.

## 1 Data

**Fig. S1:**
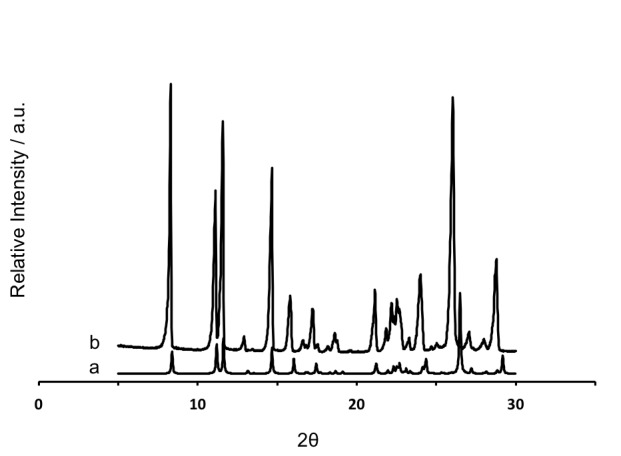
XRPD diffractograms correspondent to the 1:1 CAF:GLU system: (a) cocrystal data obtained from CSD, code EXUQUJ (form II), (b) cocrystal obtained by cooling crystallization.

**Fig. S2:**
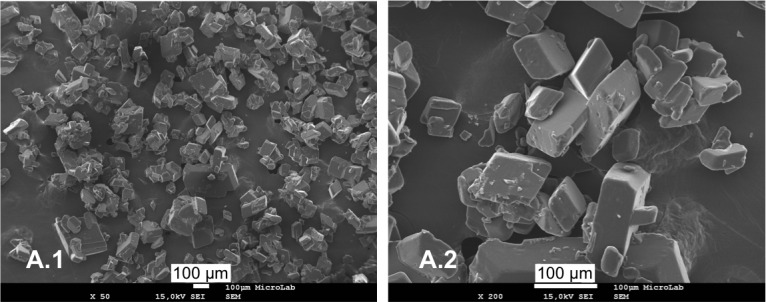
Micrographs correspondent to the 1:1 CAF:GLU cocrystal produced by cooling crystallization.

**Fig. 1:**
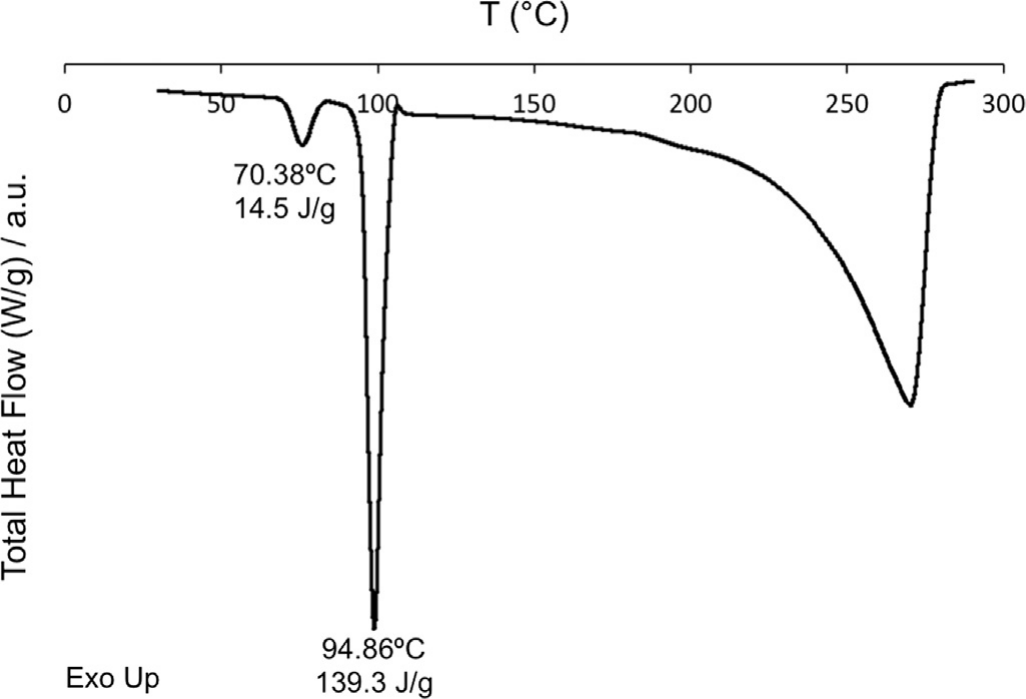
Total heat flow thermogram correspondent to pure GLU. The onset temperatures and enthalpy values associated to the endothermic events are also indicated.

**Fig. 2:**
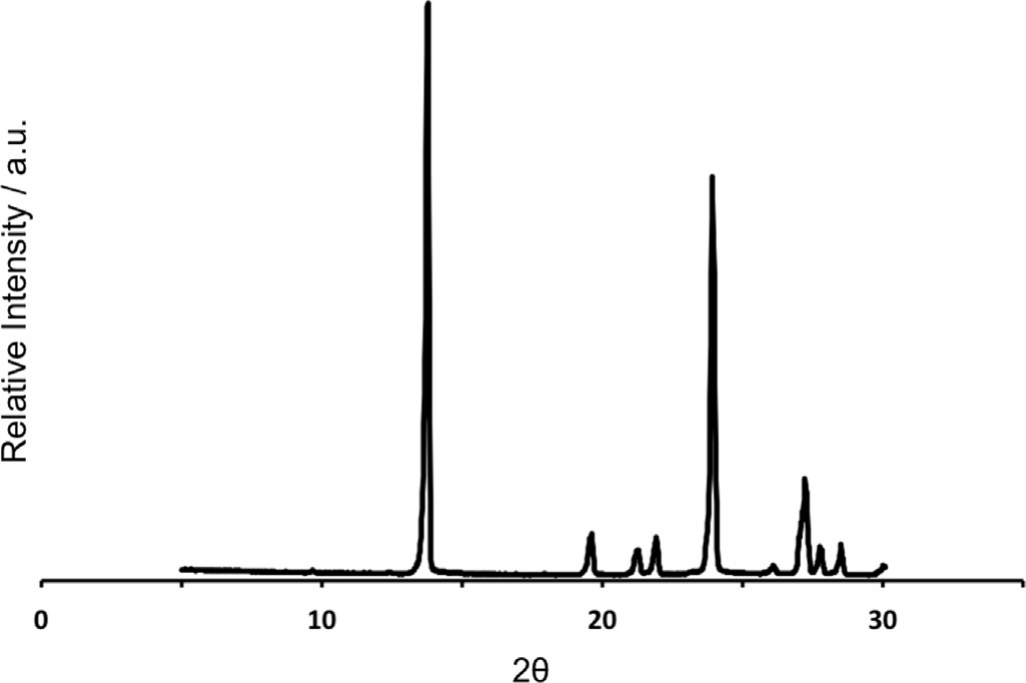
XRPD diffractogram correspondent to pure GLU.

First, the DSC thermal analysis and X-ray powder diffraction (XRPD) of pure Glutaric Acid are shown (Fig. 1, Fig. 2). Pure Glutaric Acid was used as a coformer to produce 1:1 Caffeine:Glutaric Acid cocrystals (hereafter, designated as 1:1 CAF:GLU) using spray congealing [1]. Secondly, the XRPD of the cocrystal obtained from cooling crystallization [2] is shown in Fig. S1, together with diffractogram of the same cocrystal obtained from the Cambridge Software Database (CSD). The SEM image of the cocrystal obtained is also shown (Fig. S2). Finally, the XRPD data necessary for estimating the purity of the spray-congealed cocrystals obtained in [1] is presented (Appendix A, Appendix A).

## 2 Experimental design, materials and methods

### 2.1 Thermal analysis and XRPD data of pure Glutaric Acid (GLU)

.

### 2.2 XRPD and SEM data of 1:1 CAF:GLU cocrystal produced by cooling recrystallization

The cooling crystallization method employed to produce form II of the 1:1 CAF:GLU was based on the work of Yu et al. [2]. This cocrystal served as a reference cocrystal (*i.e.* 100% pure) for the limit test applied to evaluate cocrystal purity (Section 2.3).

Fig. S1 shows the XRPD diffractogram obtained for the cocrystal obtained from cooling crystallization together with the diffractogram of the polymorphic form II of the 1:1 CAF:GLU cocrystal obtained from the Cambridge Software Database (CSD).

Fig. S2. shows the SEM images obtained for this cocrystal. Plate-shaped individual cocrystals were observed.

### 2.3 XRPD data associated with the limit test developed for the evaluation of cocrystal purity

**Fig. S3:**
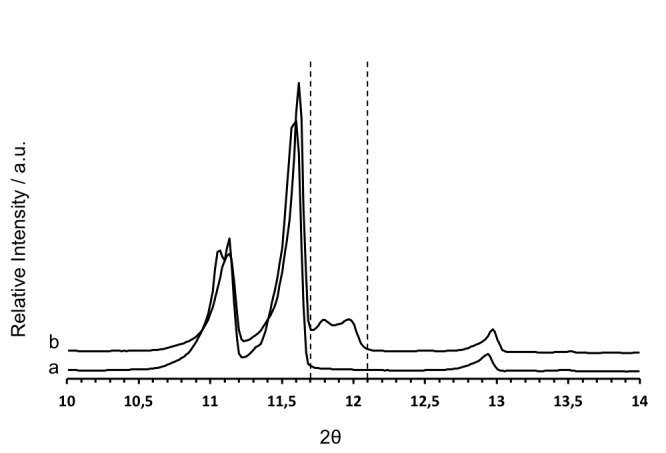
XRPD diffractograms, at slow scan, correspondent to (a) pure 1:1 CAF:GLU cocrystal produced by cooling crystallization (Section 2.2) and (b) a physical mixture of said cocrystal and 5 wt% pure CAF. The dashed lines represent the integration interval (*i.e.* 11.7–12.1 2θ).

Fig. S3 shows the XRPD diffractograms of the pure form II 1:1 CAF:GLU cocrystal produced by cooling crystallization (Section 2.2) and a physical mixture of said cocrystal and 5 wt% pure CAF. The reflection at 11.8 2θ was used as the reference for evaluating the purity of the spray-congealed cocrystals produced in [1].

**Fig. S4:**
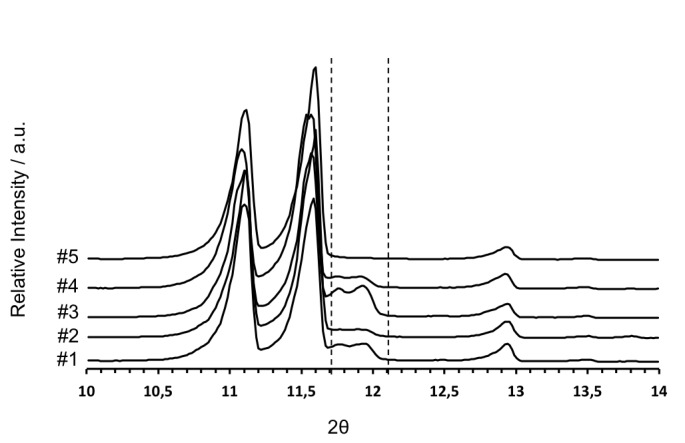
XRPD diffractograms, at slow scan, correspondent to the spray-congealed 1:1 CAF:GLU cocrystals produced in [1]: #1 to #5 – different tests performed according to the experimental design. The dashed lines represent the integration interval (*i.e.* 11.7–12.1 2θ).

Fig. S4 shows the XRPD diffractograms correspondent to the cocrystals produced by spray-congealing in [1]. Five cocrystals (Tests #1 to #5) were produced according to a design of experiments. The reflections at 11.8 2θ were integrated and the areas were compared with the area obtained from the reference (Fig. S3, diffractogram b).

## Footnotes

Appendix A Supplementary data associated with this article can be found in the online version at doi:10.1016/j.dib.2016.04.074.
